# Balance Training Under Fatigue: A Randomized Controlled Trial on the Effect of Fatigue on Adaptations to Balance Training

**DOI:** 10.1519/JSC.0000000000004620

**Published:** 2023-10-06

**Authors:** Martin Keller, Eric Lichtenstein, Ralf Roth, Oliver Faude

**Affiliations:** Department of Sport, Exercise and Health, University of Basel, Basel, Switzerland

**Keywords:** neuromuscular performance, injury prevention, sway path length, postural control

## Abstract

Keller, M, Lichtenstein, E, Roth, R, and Faude, O. Balance training under fatigue: a randomized controlled trial on the effect of fatigue on adaptations to balance training. *J Strength Cond Res* 38(2): 297–305, 2024—Balance training is an effective means for injury prevention in sports. However, one can question the existing practice of putting the balance programs at the start of a training session (i.e., train in an unfatigued state) because the occurrence of injuries has been associated with fatigue. Therefore, the aim of this study was to assess the influence of balance training in a fatigued or an unfatigued state on motor performance tested in fatigued and unfatigued conditions. Fifty-two, healthy, active volunteers (28.0 years; 19 women) were randomly allocated to 1 of 3 different training groups. The BALANCE group completed 6 weeks of balance training. The other 2 groups completed the identical balance tasks either before (BALANCE-high-intensity interval training [HIIT]) or after (HIIT-BALANCE) a HIIT session. Thus, these groups trained the balance tasks either in a fatigued or in an unfatigued state. In PRE and POST tests, balance (solid ground, soft mat, wobble board) and jump performance was obtained in fatigued and unfatigued states. Balance training resulted in reduced sway paths in all groups. However, the linear models revealed larger adaptations in BALANCE-HIIT and BALANCE when compared with HIIT-BALANCE (*d* = 0.22–0.71). These small to moderate effects were—despite some uncertainties—consistent for the “unfatigued” and “fatigued” test conditions. The results of this study revealed for the first time that balance training under fatigue results in diminished adaptations, even when tested in a fatigued state. Therefore, the data indicate that balance training should be implemented at the start of a training session or in an unfatigued state.

## Abstract 2

Supplemental Digital Content is Available in the Text.

## Introduction

Balance training is an appropriate means to notably reduce the risk of ankle injuries, particularly regarding recurrences, in athletic populations (18). For example, meta-analyses have verified the preventive effect of sensorimotor training on ankle sprains (4,31). In one of these meta-analyses, the authors not only showed a reduced injury incidence but also showed an improved dynamic neuromuscular control in the form of an improved Star Excursion Balance test performance, postural sway, and joint position sense (4). Therefore, the authors concluded that these changes in sensorimotor control after balance training might contribute to injury prevention.

Injury prevention programs (i.e., balance tasks) are commonly performed in an unfatigued state because fatigue reduces the performance of relevant measures of postural control (7,33). However, one can question the existing practice of prescribing the injury prevention programs at the start of a training session (30). There is evidence that ankle injuries occur more often at the end of the first half or at the end of a match in sports such as football, rugby, or soccer, and this has often been associated with fatigue (3). A causal relation of fatigue and injury incidence is difficult to prove for game situations, but a recent meta-analysis has shown that in laboratory setting, acute fatigue interventions negatively affect intrinsic risk factors of lower-extremity injuries (40). These studies showed compelling evidence for not only fatigue-induced reductions in reflexes (1,28) but also impaired dynamic joint control (1,6,8,28), making fatigued athletes more prone to injuries.

Based on the idea that fatigue is associated with an enhanced injury risk because of diminished neuromuscular control (e.g., reduced joint stability), some authors recommended to consider the concept of “training under fatigue” for injury prevention programs (30). The idea of considering “training under fatigue” is based on the principle of training specificity known from motor control (15) and also from exercise science (34). The theory of training specificity stipulates that adaptations to a training intervention are highly specific with little or no transfer to other (unrelated) motor tasks (22) and physiological states (34). For example, Small et al. (34) investigated the effect of timing of nordic hamstring exercises during soccer training. The authors asked a first group of volunteers to train nordic hamstring exercises during the warm-up of soccer training, whereas a second group performed the identical tasks during the cooldown. Despite an identical training load during the intervention period, the 2 groups showed different training adaptations in eccentric hamstring peak torque. Before the intervention, both groups showed declines in peak torque after a soccer-specific fatigue protocol. After the intervention period, the warm-up group still showed this fatigue-induced decline, whereas the cooldown group showed comparable force levels with and without fatigue. However, only the warm-up group showed enhanced maximum force when subjects were tested in a unfatigued state. Therefore, these results show fatigue-dependent adaptations to resistance training with training performed under fatigue leading to better performances in a fatigued state. In line with these data and the idea of training specificity (15,19), it could also be argued that balance training should be conducted at the end rather than at the start of a training session to improve dynamic neuromuscular control in a fatigued state. There is only one study available that investigated the influence of timing of balance training on stance stability (10). In this study, one group trained the balance task before a soccer training session, whereas the other group trained the same tasks after the training session. Although both groups improved over time, the authors did not analyze whether the improved postural stability was specific to the physiological state (fatigued vs. unfatigued).

Therefore, the aim of this study was to assess the influence of balance training conducted either in a fatigued or unfatigued state on neuromuscular performance when fatigued and unfatigued. We hypothesized that balance training would result in adaptations specific to the physiological state (fatigued vs. unfatigued). Therefore, balance training under fatigue should improve performance in the fatigued state, with little or no transfer to the unfatigued state (and vice versa). Assessing a potential interaction between fatigue and the effectiveness of balance training seems relevant for preventive purposes such as injury prevention programs in team sports.

## Methods

### Experimental Approach to the Problem

**Figure 1.**
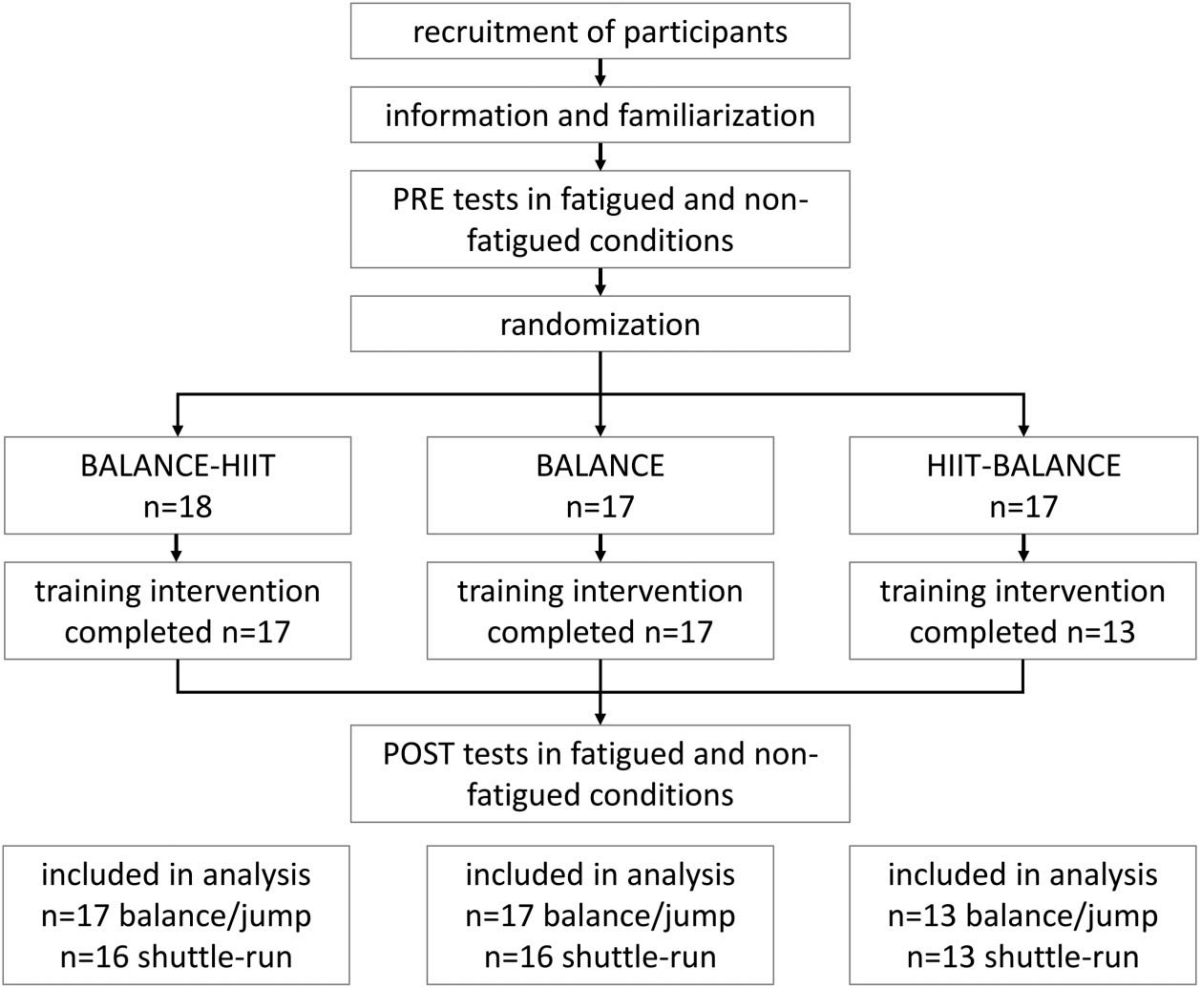
Experimental approach to the problem.

The study was a 3-armed randomized controlled trial with 1 group performing “balance training” (BALANCE), a second group participating in “balance training under fatigue” after a high-intensity interval training [HIIT] session (group HIIT-BALANCE), and a third group completing “balance training before fatigue” (BALANCE-FATIGUE). The BALANCE group served as control group because this group followed a classical balance training regimen. The reason for including BALANCE-HIIT was that we wanted to include a group with an identical metabolic and cardiovascular training load as the HIIT-BALANCE group because the HIIT protocol may affect motor learning (32,39). The general study design is depicted in Figure 1.

### Subjects

A total of 52 healthy active volunteers participated in this study. Subjects were recruited from local sport clubs. All subjects trained 1–3 hours per weeks in sports such as soccer, volleyball, combat sports, and athletics before the Covid lockdown. However, as a result of the national Covid measures, subjects were less active during the 10 weeks before the start of the study because sports facilities were closed. All subjects were informed about the general study idea but were blind to the specific study aims. Subjects had to sign an informed consent form before inclusion. The study protocol was approved by the local ethics committee (Ethikkommission Nordwest-und Zentralschweiz BASEC-ID 2019-0202). All measurements were in accordance with the guidelines from Good Clinical Practice and were in line with the Declaration of Helsinki. Volunteers were included if they were 18–38 years old and did not participate in balance training in the 12 months before the start of the study. An exclusion criterion was a history of illness or injury that could have affected testing or training.

### Procedures

#### PRE Assessments

All volunteers attended an information session at the beginning of the study. In this session, all methods and training details were explained, and the subjects practiced the balance and jumping tests. Directly after attending this general information session, the subjects completed a shuttle run test until exhaustion. After at least 24 hours, neuromuscular performance was tested (see details about measurements further below). The neuromuscular performance was recorded before and after a fatigue protocol. Fatigue was induced with a HIIT.

#### Group Allocation

The performance values obtained in PRE were used to randomly allocate the subjects to 1 of the 3 intervention groups. An Excel spreadsheet that reduces differences between the group means of subject characteristics was used for group allocation (16). We used the parameters such as age, gender, and achieved PRE performances (shuttle run test, jump height, balance performance in fatigued and unfatigued states) to minimize baseline differences between the groups (HIIT-BALANCE: 29.0 ± 4.2 years, 11 men and 6 women; BALANCE-HIIT: 27.4 ± 3.1 years, 11 men and 7 women; BALANCE: 27.5 ± 4.1 years, 11 men and 7 women). Subjects could not be blinded with respect to the intervention but were blinded with respect to the aims of the study. As a result of the national Covid measures, the number of people involved in training and testing was minimized. Therefore, all tests were performed by researchers who were aware of the group assignment.

#### Intervention Period

All 3 groups performed the identical balance training intervention. The intervention lasted for 6 weeks with a total of 12 training sessions. The volunteers trained twice a week with at least 48 hours between training sessions. Subjects trained on 4 different devices: wobble board, tilt board, soft mat, and air cushion. The subjects performed 4 trials of 20 seconds on each device and leg leading to a total of 32 trials per session. To avoid fatigue, 25 seconds of rest were given between 2 consecutive trials. There was a rest period of approximately 1 minute between the devices, resulting in a duration of approximately 30 minutes per session. All sessions were supervised. The difficulty of the balance tasks was continuously adjusted to the individual and actual performance level. At the beginning of the training, all subjects performed the balance tasks with eyes open (Level 1). If the tasks of level 1 could be solved without problems, the subjects were asked to train the next difficulty level (level 2: eyes open with motor or cognitive dual tasks such as counting backward or catching or passing a ball; level 3: eyes closed; level 4 eyes closed with motor or cognitive dual tasks). The different levels allowed an individualized and continuous progression of the task difficulty during the intervention period.

Although the BALANCE group trained balance tasks only, the other 2 groups additionally completed a fatigue protocol either before or after the balance tasks. In the HIIT-BALANCE group, the fatigue protocol was performed before the balance exercises and thus corresponded to a “balance training under fatigue.” By contrast, the BALANCE-HIIT group trained first the balance tasks and directly afterwards the fatigue protocol. Thus, the acute fatigue could not have influenced balance performance in this group. The fatigue protocol was a HIIT session with subjects running on an athletics track. Each HIIT session involved 4*4 minutes of high-intensity intervals with a heart rate above >90% of the individual maximum heart rate, interspersed with 3 minutes of active recovery (heart rate ∼60% of maximum heart rate).

#### POST Assessments

Following the intervention, measures of performance were obtained in POST tests. The POST assessments were 2–5 days after the last training session. The data assessment was identical with the PRE assessments to allow for PRE to POST comparisons.

#### Dropouts and Lost Data

Five subjects did not complete the whole intervention period because of health issues that were not related to the study (*n* = 1), limited time because of unexpectedly high workloads in job (*n* = 1), problems in school (*n* = 2), and other personal reasons (*n* = 1). Thus, from the 52 recruited subjects, only 47 were included in the final analyses. From these 47 subjects, 2 were quarantined (possible infection with coronavirus) the day after the balance and jump assessments and could not complete the shuttle run test after the intervention period. Therefore, the data of 47 subjects are included in the final analysis of the balance and jump performance assessments, whereas only 45 data sets are included in the shuttle run analysis.

#### Measurements

In PRE and POST assessments, neuromuscular performance was assessed before and after a fatigue protocol. The fatigue protocol was a single HIIT session that started 3 minutes after the end of the assessments in the unfatigued state. The evaluation of performance in the fatigued state started immediately after the end of the fatigue protocol. The order of testing conditions (balance and jumping tests) was randomized between the subjects but was kept identical for each subject in PRE and POST assessments.

### Balance Assessments

Balance performance was measured while subjects stood on the right leg. The sway path was assessed while subjects stood on solid ground, while balancing on a soft mat (Balance-pad, Airex), and while standing on a balance wobble board (Kübler Sport; 37*9 cm; radius of sphere 13.4 cm; height of sphere: 7.8 cm). The overall sway path length (in millimeters) was measured in all conditions using a force plate (Leonardo Mechanograph GRFP LT; Novotec Medical, Pforzheim, Germany). The sway path length is defined as the common length of the trajectory of the center of pressure and was calculated as the sum of the point-to-point Euclidean distances. All balance assessments were accomplished according to standard operating procedures established in our laboratory. We recorded 3 trials per device with a duration of 20 seconds. Subjects were asked to rest for 10 seconds between 2 trials. No rest was given between the conditions. The resting time was minimized because measurements were to be performed in a short time after the fatigue protocol.

### Jump Height

Subjects performed 3 countermovement jumps in the fatigued and unfatigued states. Subjects were asked to start the movement in an upright standing position, then to drop down to a volitional depth and to jump as high as possible. All jumps were performed with the hands held akimbo to limit any upper-extremity influence on the jumps. Three maximum jumps were recorded in the unfatigued state and in the fatigued state. Vertical jump performance was analyzed using a calibrated force plate (Leonardo Mechanograph GRFP LT; Novotec Medical). Jump height was calculated from takeoff velocity through the impulse momentum method. The mean of the 3 jumps per physiological state (unfatigued vs. fatigued) was used for data analysis.

### Shuttle Run

The shuttle run test was used to monitor the endurance performance of the subjects before and after the intervention period. The test has a high test-retest reliability that allows valid predictions of V̇o_2_max (23). During the test, subjects ran back and forth over a 20-m track, with the initial speed (8.0 km·h^−1^) being increased by 0.5 km·h^−1^ every minute. When the end of the track had to be reached, the pace was indicated by a loud beep. If a subject was unable to reach the end of the 20-m track at the time of the beep for 2 consecutive repetitions, the individual maximum speed was considered to have been reached, and the test was stopped. The number of completed 20-m repetitions was counted and used for data analysis. Shuttle run performance was tested on separate days in PRE and POST assessments with at least 24 hours of rest from neuromuscular performance testings. Subjects were verbally encouraged to perform to their best capacity.

### Statistical Analyses

For the comparison of adaptations between the groups, we calculated mixed linear models with the outcome of a measurement (e.g., jump height) as the dependent variable and time (PRE vs. post), group (BALANCE vs. HIIT-BALANCE vs. HIIT-BALANCE), and physiological state (fatigued vs. unfatigued) as factors. Separate analyses were calculated for each outcome (shuttle run performance, jump height, sway path while standing on (a) solid ground, (b) balance wobble board, (c) soft mat). Random intercepts and slopes on the individual level were used. This allowed for analyzing different trajectories in the adaptations of postural stability, shuttle run performance, and jump height over time between the groups accounting for individual variation. All estimates of the interaction between time and group are reported together with the corresponding standardized effect size and 95% confidence interval. Thus, the reported estimates can be interpreted as the difference in performance changes between the groups over time. As recommended for longitudinal studies, any training adaptations are also reported using change scores (13). The effect sizes are discussed in terms of negligible (<0.2), small (0.2–0.5), moderate (0.5–0.8), and large (≥0.8) effects (2).

## Results

### Balance Wobble Board

**Figure 2.**
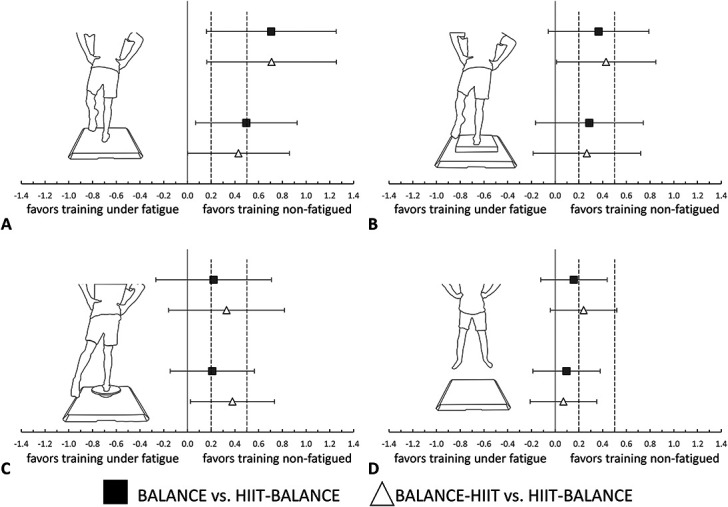
The comparisons of group-specific adaptations are shown with HIIT-BALANCE serving as reference group in the statistical model. All subjects were tested before and after the training intervention (PRE vs. POST) while balance on 3 different balance devices. Data obtained while standing on solid ground are shown in (A), data from the soft mat in (B), and results from the wobble board in (C). Jump height adaptations are illustrated in (D). All comparisons are shown for unfatigued (upper part of A–D) and fatigued (lower part of A–D) conditions. In (A–D), the comparison of BALANCE vs. HIIT-BALANCE (black squares) and BALANCE-HIIT vs. HIIT-BALANCE (white diamonds) are illustrated. Dashed vertical lines indicating trivial (0–0.2), small (>0.2), moderate (>0.5), or large (>0.8) effects included to help interpret the results. HIIT = high-intensity interval training.

After the intervention period, reduced sway path lengths were found when subjects were tested on the balance wobble board (Table 1 and Figure 2). On average, BALANCE (unfatigued: −13.6%; fatigued: −6.5%) and BALANCE-HIIT (unfatigued: −18.7%; fatigued: −10.4%) showed higher reductions than HIIT-BALANCE (unfatigued: −8.4%; fatigued: +0.6%). The effect size of the interaction effects (Table 1) indicated that the different training adaptations can be interpreted as small effects, but small negative to large positive effects are also compatible with the data (Figure 2). All individual adaptations can be found in the Supplemental Digital Content 1 (see Supplementary Materials, http://links.lww.com/JSCR/A412).

**Table 1:** The results of the assessments obtained in PRE and POST measurements.*†

	BALANCE			BALANCE-HIIT			HIIT-BALANCE			Mixed model analysis		
	PRE	POST	Δ (95% CI)	PRE	POST	Δ (95% CI)	PRE	POST	Δ (95% CI)	BALANCE vs. HIIT-BALANCE	BALANCE-HIIT vs. HIIT-BALANCE	BALANCE vs. BALANCE-HIIT
Unfatigued												
Solid ground (mm)	707 ± 155	621 ± 71	−86 (−139; −34)	676 ± 147	590 ± 133	−86 (−125; −48)	650 ± 162	672 ± 250	22 (−63; 107)	*B* = −108.1; *p* = 0.015; *d* = −0.71	*B* = −108.5; *p* = 0.015; *d* = −0.71	*B* = 0.472; *p* = 0.991; *d* = 0.003
Wobble board (mm)	1,097 ± 287	917 ± 152	−180 (−293; −69)	1,099 ± 338	884 ± 260	−215 (−301; −129)	1,057 ± 316	944 ± 251	−113 (−222; −3)	*B* = −68.0; *p* = 0.378; *d* = −0.22	*B* = −102.3; *p* = 0.188; *d* = −0.33	*B* = 34.2; *p* = 0.633; *d* = 0.11
Soft mat (mm)	876 ± 176	740 ± 135	−136 (−181; −90)	859 ± 240	709 ± 182	−150 (−198; −100)	845 ± 271	792 ± 216	−53 (−158; 51)	*B* = −82.1; *p* = 0.097; *d* = −0.37	*B* = −95.4; *p* = 0.055; *d* = −0.43	*B* = 13.3; *p* = 0.769; *d* = 0.06
Jump height CMJ (cm)	38.4 ± 8.9	38.8 ± 7.6	0.4 (−1.0; 1.9)	39.8 ± 8.0	40.9 ± 7.3	1.1 (−0.4; 2.6)	40.6 ± 5.7	39.8 ± 6.4	−0.8 (−2.2; 0.6)	*B* = 1.2; *p* = 0.273; *d* = 0.16	*B* = 1.83; *p* = 0.103; *d* = 0.24	*B* = −0.6; *p* = 0.553; *d* = −0.8
Shuttle run (20-m shuttles)	83 ± 37	84 ± 35	1 (−3; 5)	87 ± 24	96 ± 24	9 (7; 12)	88 ± 20	97 ± 16	9 (4; 14)	*B* = −7.7; *p* = 0.015; *d* = −0.28	*B* = 0.3; *p* = 0.927; *d* = 0.01	*B* = −7.9; *p* = 0.008; *d* = −0.29
Fatigued												
Solid ground (mm)	677 ± 115	651 ± 84	−26 (−65; 13)	653 ± 137	636 ± 155	−17.1 (−45.7; 11.4)	628 ± 170	670 ± 167	42 (−14; 99)	*B* = −68.6 *p* = 0.028; *d* = −0.50	*B* = −59.4; *p* = 0.055; *d* = −0.43	*B* = −9.141; *p* = 0.747; *d* = −0.66
Wobble board (mm)	940 ± 151	876 ± 164	−64 (−113; 14)	1,010 ± 292	905 ± 281	−105 (−142; −68)	918 ± 268	904 ± 237	−14 (−106; 79)	*B* = −50.5; *p* = 0.254; *d* = −0.22	*B* = −91.6; *p* = 0.042; *d* = −0.38	*B* = 41.1; *p* = 0.318; *d* = 0.17
Soft mat (mm)	843 ± 155	763 ± 112	−80 (−144; −16)	845 ± 249	769 ± 225	−76 (−100;−40)	817 ± 217	797 ± 181	−20 (−114; 74)	*B* = −59.4; *p* = 0.219; *d* = −0.29	*B* = −55.0; *p* = 0.255; *d* = −0.27	*B* = −4.5; *p* = 0.92; *d* = −0.02
Jump height CMJ (cm)	37.7 ± 8.6	38.9 ± 8.2	1.2 (0.2; 2.1)	40.1 ± 7.7	41.0 ± 7.2	0.9 (−0.9; 2.8)	40.2 ± 5.9	40.7 ± 6.9	0.4 (−0.7; 1.6)	*B* = 0.7; *p* = 0.498; *d* = 0.10	*B* = 0.5; *p* = 0.62; *d* = 0.07	*B* = 0.195; *p* = 0.84; *d* = 0.03

* CI = confidence interval; HIIT = high-intensity interval training; *B* = estimator; *d* = standardized estimates of the linear mixed-effects models.

† Data are illustrated as means with *SD*s for PRE and POST values. Differences from PRE to POST are shown as standardized mean difference with 95% CI. Results of the linear mixed models are shown to illustrate differences between the groups over time.

### Soft Mat

On average, all groups improved in balance performance from PRE to POST in the soft mat balance task (mean values, *SD*s and change scores in Table 1). On the soft mat, reduced COP sway path lengths were found for BALANCE (−14.5%), BALANCE-HIIT (−16.8%), and HIIT-BALANCE (−3.7%) when assessments were done in the unfatigued state. When assessments were done in the fatigued state, slightly lower adaptations were found for BALANCE (−7.9%), BALANCE-HIIT (−8.7%) and HIIT-BALANCE (0.1%). Regarding between-group differences in change scores, the effect sizes indicate that BALANCE and BALANCE-HIIT show enhanced reductions in sway path lengths when compared with HIIT-BALANCE (Figure 2). However, the data are also compatible with negative negligible to large positive effects. All individual adaptations can be found in the Supplemental Digital Content 1 (see Supplementary Materials, http://links.lww.com/JSCR/A412).

### Solid Ground

When tested in the unfatigued condition, the sway path lengths while standing on solid ground decreased on average for BALANCE (−9.9%) and BALANCE-HIIT (−12.4%) but not for HIIT-BALANCE (+3.3%). Less pronounced average effects were observed in the fatigued testing condition with reductions for BALANCE (−2.7%) and BALANCE-HIIT (−3.0%) but increased sway path lengths for HIIT-BALANCE (+9.0%). The group*time interaction showed moderate differences for BALANCE vs. HIIT-BALANCE and for BALANCE-HIIT vs. HIIT-BALANCE in the unfatigued testing condition—because of the confidence intervals of the effect sizes, the data are also compatible with negligible to large effects (Figure 2). In the fatigued testing condition, small differences were observed for the same comparisons between the groups with confidence intervals ranging from negligible to large effects. All individual adaptations can be found in the Supplemental Digital Content 1 (see Supplementary Materials, http://links.lww.com/JSCR/A412).

### Shuttle Run Performance

**Figure 3.**
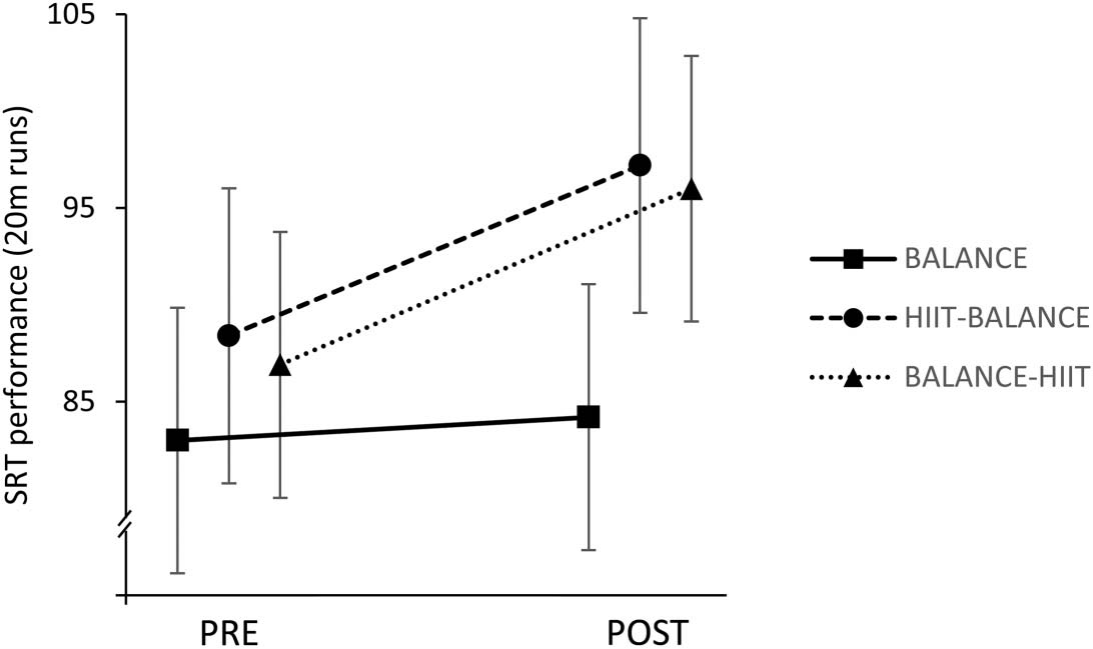
Average obtained from shuttle run performances in the PRE and POST assessments. Both groups that trained HIIT during the intervention increased the number of shuttles by approximately 12%, whereas the BALANCE group did not show relevant improvements (+3.6%). HIIT = high-intensity interval training.

The shuttle run data revealed that subjects from BALANCE-HIIT (PRE: 87 ± 24 shuttles; POST: 96 ± 24 shuttles) and HIIT-BALANCE (PRE: 88 ± 20 shuttles; POST: 97 ± 16 shuttles) clearly improved their endurance capacity, whereas BALANCE showed nearly constant performance (PRE: 83 ± 37 shuttles; POST: 84 ± 35 shuttles). When comparing the training adaptations between the groups, the mixed linear model revealed higher gains with a small effect for HIIT-BALANCE and BALANCE-HIIT when compared with BALANCE (Table 1 and Figure 3), but the data are also compatible with negligible to medium effects.

### Jump Performance in Unfatigued Condition

Only minor changes from PRE to POST were identified for jump height. The fixed effects parameter estimates (Table 1) showed slightly higher adaptations for BALANCE and BALANCE-HIIT when compared with HIIT-BALANCE. In terms of effect sizes, these differences can be interpreted as negligible and small, respectively. However, because of the large confidence intervals of these effect sizes, the data are also in line with negligible negative to large positive effects (Figure 2).

### Jump Performance in Fatigued Condition

The adaptations in jump height were similar between the groups when subjects jumped in a fatigued state. When comparing the PRE with POST adaptations between the groups, the mixed linear model revealed negligible differences in the group-specific adaptations (Table 1 and Figure 2). The confidence intervals of the effect sizes show that the data are also compatible with small negative to small positive effects.

## Discussion

In this study, we assessed postural stability, shuttle run performance, and jump height before and after a balance training intervention. All subjects performed the identical balance training, whereas the time before and after the balance training sessions varied between the groups. One group (HIIT-BALANCE) trained the balance tasks in a fatigued state, the other 2 groups (BALANCE and BALANCE-HIIT) in a unfatigued state. Our results on postural stability show that balance training under fatigue results in diminished improvements in postural sway compared with balance training in an unfatigued state, both when tested in the unfatigued and in fatigued states. This effect has been observed for all testing conditions, but with the effect being greatest when subjects were tested on the solid surface.

The idea of considering “training under fatigue” is based on theoretical assumptions (30) and also on empirical evidence (34). In line with these data, we expected fatigue-specific adaptations, with higher training gains in the HIIT-BALANCE group for tests in the fatigued state and higher adaptations in BALANCE and BALANCE-HIIT when subjects were unfatigued. However, the results of our balance tests are not in line with our hypothesis. HIIT-BALANCE showed consistently lower training adaptations compared with the groups that trained in the unfatigued state, although effects are accompanied with uncertainty because of large confidence intervals. Nevertheless, BALANCE and BALANCE-HIIT consistently showed higher adaptations compared with HIIT-BALANCE in both the fatigue and unfatigue tests; these effects were most pronounced when subjects were measured on solid ground while the confidence intervals of the effect sizes for measurements in unstable conditions also include small negative effects. These differences in the magnitude of adaptations might be explained by task complexity that was—based on sway path lengths—lowest on solid ground. Despite the fact that the magnitude of the effect differed between testing conditions, the acquired data were not in line with our hypothesis and we cannot provide any definite explanation. One reasonable explanation is that the HIIT protocol resulted in a lower training quality in HIIT-BALANCE resulting in diminished training gains. There is indeed evidence supporting the idea that acute local fatigue results in increased postural sway (7,33). It has also been summarized that not only local but also general fatigue results in increased postural sway (29). The authors of this review highlighted that endurance tasks that are above the lactate accumulation threshold or are prolonged have the potential to decrease balance performance. Although we did not measure lactate values in our subjects, HIIT training sessions with intensities >90% of the individual maximum heart rate typically result in increased blood lactate concentrations. Therefore, the training load applied during HIIT most likely induced fatigue, resulting in an altered effectiveness of sensory inputs and motor output (29). Because studies have also shown fatigue-related reductions of voluntary activation (20) and inhibition of extensor muscles by fatigue-sensitive afferents (26), training responses may have been generally attenuated in the HIIT-BALANCE group. Another possible explanation is the motor task selected. While Small et al. (34) assessed the training under fatigue phenomenon for strength tasks, we examined the phenomenon for postural control. Therefore, it should be noted that neural adaptations differ between balance and strength training. For example, it has recently been observed that balance training results in increased levels of short-interval intracortical inhibition, whereas reduced levels of intracortical inhibition have been found after strength interventions (36). Thus, the distinct brain regions involved in the control of these motor tasks and the neural adaptations to training are different (17,37). Therefore, these differences in the neural control and neural adaptations could also explain why we did not find enhanced adaptations after balance training under fatigue.

Several previous studies reported a positive effect of balance training on the rate of force development (12) or jump height (11,38). Therefore, we assessed jump height performance before and after the intervention period when subjects were fatigued and unfatigued. Changes from PRE to POST and adaptations between the groups did not show any relevant differences. Based on the acquired data, we cannot explain why we and others (9) did not find enhanced jump heights after the balance intervention. Therefore, future studies need to identify factors with the potential to explain whether and when balance training results in jump height improvements.

We monitored endurance capacity before and after the intervention period. As expected, an enhanced endurance performance was found for HIIT-BALANCE and BALANCE-HIIT when compared with BALANCE. The observed improvements (>10% in both HIIT groups) show that the HIIT intervention was an effective means to promote endurance performance. This observation is well in line with several previous studies reporting similar adaptations to HIIT (14,27,35).

Endurance exercise does not only improve endurance performance but also has the potential to improve balance performance in healthy young to middle-aged adults (5) or to improve human memory formation (32,39). Based on this knowledge, we designed our study as a three-armed, randomized, controlled trial with 2 groups (BALANCE-HIIT and HIIT-BALANCE) participating in balance and endurance training and a third group (BALANCE) that did not train endurance. Therefore, the comparison of BALANCE and BALANCE-HIIT allows conclusions about the influence of endurance exercise on the learning of complex balance tasks. Considering the observed improvements in postural control, we found comparable adaptations for those 2 groups. Thus, the endurance training did not foster balance improvements in the BALANCE-HIIT group. This effect is in contrast to previous studies in which pairing endurance exercise with subsequent balance training resulted in enhanced balance learning when compared with balance training without prior cardiovascular training (24,25). These previous studies used a sequential order of endurance training followed by balance training with the idea that long-term endurance training primes the molecular machinery for neuroplasticity and facilitates subsequent motor learning (39). Unlike these previous studies, subjects in the BALANCE-HIIT group completed the balance and endurance tasks within the same training period. Thus, the impact of the HIIT sessions in optimizing molecular mechanisms supporting motor learning was very likely limited. Nevertheless, the comparable adaptations of BALANCE-HIIT and BALANCE are highly relevant because this comparison reveals that the additional load induced by HIIT did not cause any interfering or facilitating effect on the balance outcomes. Therefore, it can be concluded that training under fatigue actually caused the reduced training adaptations observed in HIIT-BALANCE.

Despite great efforts in creating a solid study design, this study has some limitations. One limitation is that we could not randomize the testing sessions (fatigued vs. unfatigued). Because all measurements started with the unfatigued measurements, there could have been an order effect. In addition, we did not measure balance performance (i.e., sway paths) during the training sessions. Therefore, we do not know if balance performance was actually impaired during training when a HIIT protocol was performed before the balance tasks. Measuring indicators of fatigue such as rating of perceived exhaustion during assessments and trainings would also have been helpful in assessing the consistency of fatigue. However, these minor limitations do not have any influence on our research question—the question if “balance training under fatigue” should be recommended when aiming for a high neuromuscular performance in fatigued conditions. Based on our data, we can conclude (with some uncertainty because of the large confidence intervals) that training balance tasks in an unfatigued state results in enhanced training gains when compared with balance training under fatigue. However, these data on balance and jump do not allow any definite conclusions about the influence on injury risks. Therefore, future studies need to address the question whether and how these effects affect injury incidences of e.g., ankle sprains. Other minor limitations are that an inactive control group was not included and that no information was collected on the menstrual cycle. Acquiring this information would have been helpful for the interpretation of the balance adaptations because the menstrual cycle can have an influence on the neuromuscular control of ankle-stabilizing muscles (21).

**Practical Applications**

A previous study provided theoretical arguments explaining why training under fatigue might be theoretically superior than training in a unfatigued state (30). However, no previous study has collected empirical data to answer this question. Therefore, this study shows for the first time that balance training under fatigue does not result in superior performance when compared with balance training in an unfatigued state. In contrast to our hypothesis, this effect has been observed through assessments in the fatigued and unfatigued states. Therefore, it can be concluded that the existing practice of putting training balance tasks at the beginning of a training sessions (i.e., in an unfatigued state) is the more efficient way to improve balance performance.

## Supplementary Material

SUPPLEMENTARY MATERIAL
