# Supplementary material for: Balance Training Under Fatigue: A Randomized Controlled Trial on the Effect of Fatigue on Adaptations to Balance Training
Source: J Strength Cond Res. 2023 Oct 6;38(2):297–305. doi: 10.1519/JSC.0000000000004620 (PMC10798588; doi:10.1519/JSC.0000000000004620)
Supplement: SUPPLEMENTARY MATERIAL [file jscr-38-297-s001.pptx]

## Slide 1
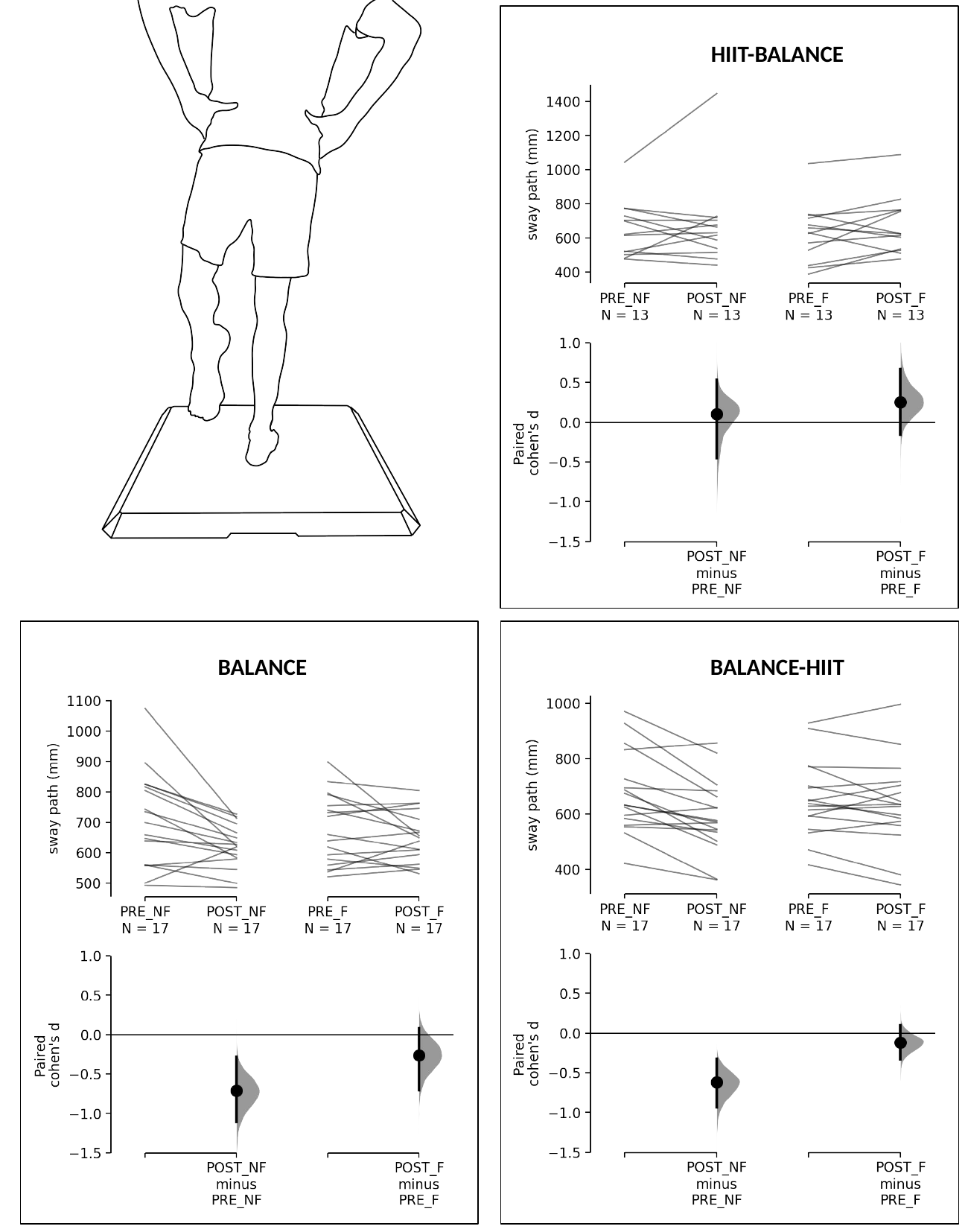

HIIT-BALANCE
BALANCE
BALANCE-HIIT

## Slide 2
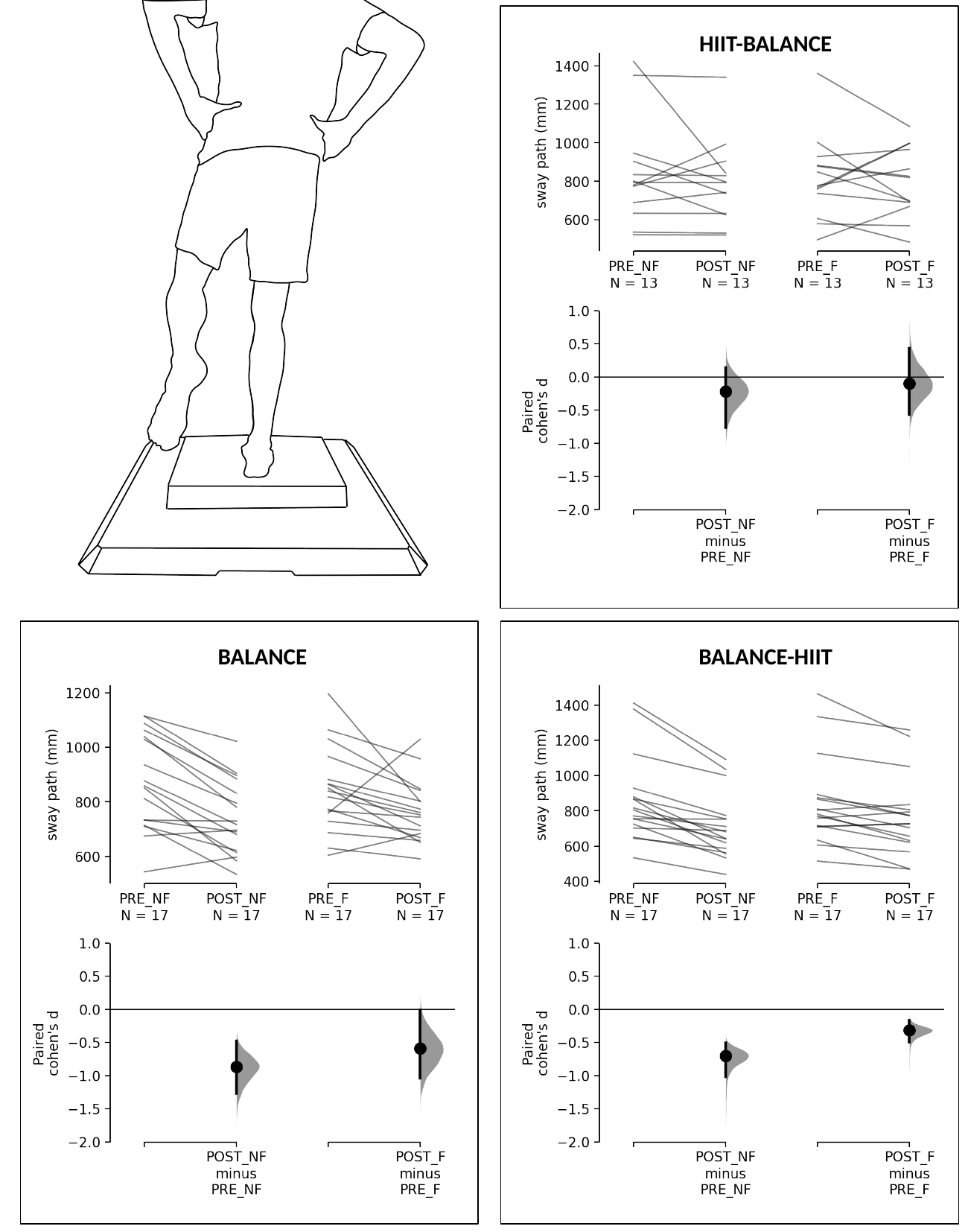

HIIT-BALANCE
BALANCE
BALANCE-HIIT

## Slide 3
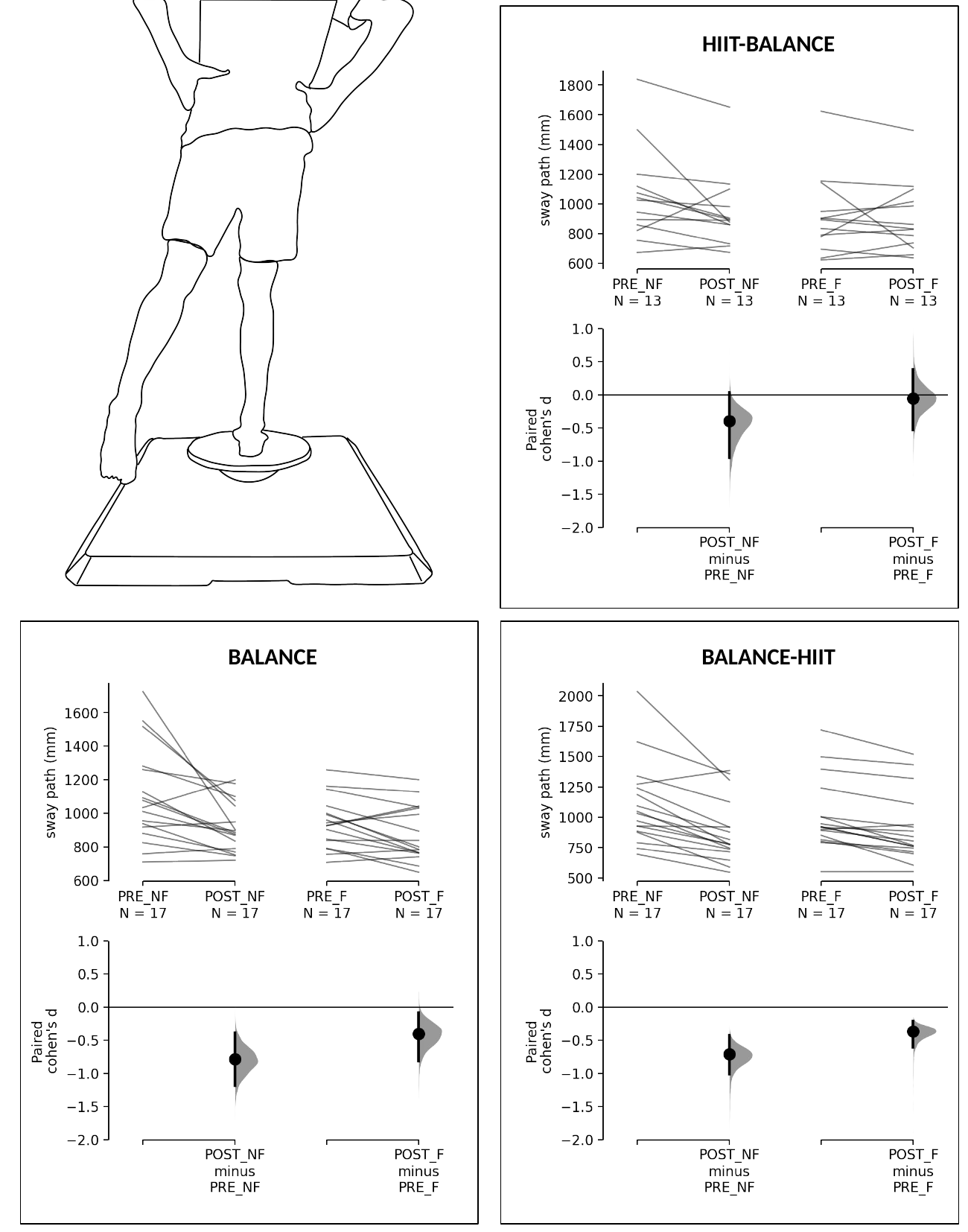

HIIT-BALANCE
BALANCE
BALANCE-HIIT
